# Supplementary material for: A Y chromosome-linked genome editor for efficient population suppression in the malaria vector Anopheles gambiae
Source: Nat Commun. 2025 Jan 2;16:206. doi: 10.1038/s41467-024-55391-8 (PMC11696527; doi:10.1038/s41467-024-55391-8)
Supplement: Supplementary file 1 — Supplementary Information [file 41467_2024_55391_MOESM1_ESM.pdf]

SUPPLEMENTARY INFORMATION

SUPPLEMENTARY FIGURES

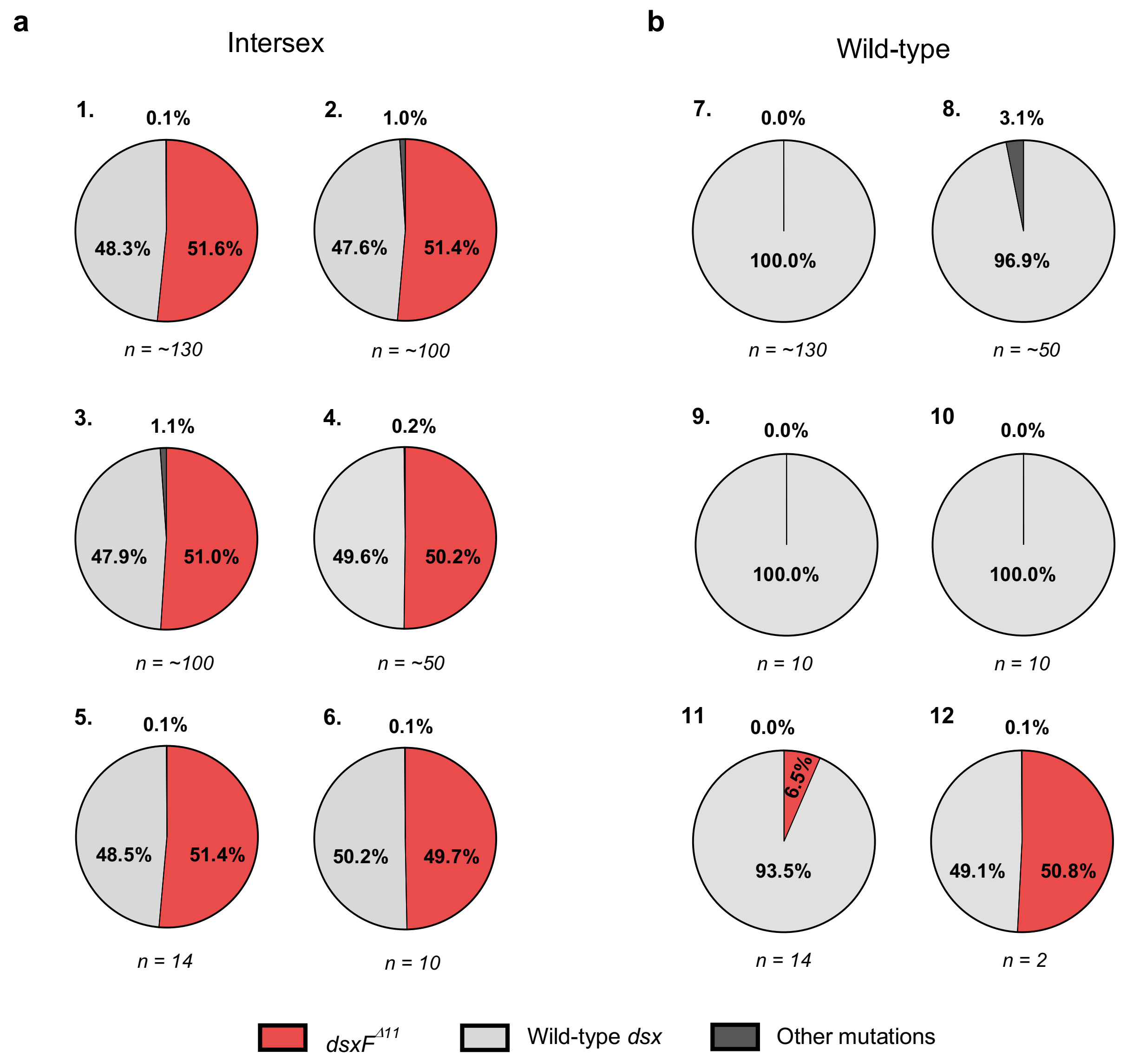

Supplementary figure 1 – Allelic frequency in pools of female individuals showing intersex and wild-type phenotypes in the progeny of YLE^{dsx} males. Female individuals in the progeny of YLE^{dsx} males were pooled according to their phenotype at different generations during the maintenance of the YLE^{dsx}-A and YLE^{dsx}-B strains. Amplicon sequencing of the *dsx* locus was then performed and analysed. Each pie chart shows the frequency of the alleles found in a pool of individuals of size *n* (indicated below the chart). (**a**) Pools of intersex individuals always contained the *dsxF*$^{\Delta 11}$ and the wild-type *dsx* alleles in frequencies close to 50%. In the pool number 2, a one-base pair deletion located within the coding sequence of exon 5 was found at 1%, which would correspond to two heterozygous individuals for this mutation. In the pool number 3, the same one-base pair deletion was found at 0.5% frequency, as well as a nine-base pair deletion also located within the coding sequence of exon 5 found at 0.6%, each of them equivalent to one heterozygous individual in the pool. (**b**) Pools of wild-type looking individuals contained mainly the wild-type *dsx* allele. In pool number 8, a six-base pair insertion within the coding sequence of exon 5 was found. The *dsxF*$^{\Delta 11}$ allele was found in a frequency corresponding to two heterozygous individuals in the pool number 11, and in the two individuals in the pool number 12 (representing ~1% of the total reads among the different pools of wild-type females).

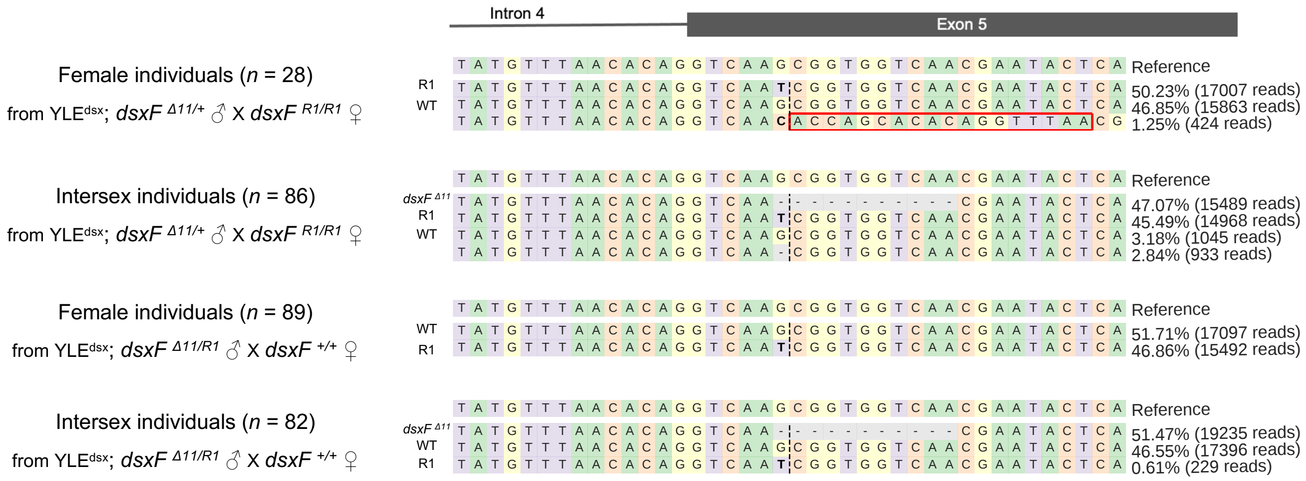

Supplementary figure 2 – Allelic frequency in the pools of female and intersex individuals from the progeny of the two crosses performed with the functional and uncleavable *R1* allele. Amplicon sequencing of the *dsx* locus was performed in pools of female and intersex individuals from the progeny of YLE^{dsx}; $dsxF^{\Delta 11/+}$ males and $dsxF^{R1/R1}$ females (two top blocks), and from the progeny of YLE^{dsx}; $dsxF^{\Delta 11/R1}$ males and $dsxF^{+/+}$ (wild-type) females (two bottom blocks). The $dsxF^{\Delta 11}$ allele was never found in the pools of female individuals, which always had the *R1* allele in heterozygosity; while this $dsxF^{\Delta 11}$ allele was always found in heterozygosity in intersex individuals with either the *R1* allele (first cross) or the wild-type *dsx* allele (second cross). An 18-base pair insertion in exon 5 was found in the pool of female individuals (red box), suggesting that this might be a recessive mutation. A one-base pair deletion within the coding sequence of exon 5 was found in the pool of intersex individuals, indicating that this may be a dominant mutation. Intron 4 and exon 5 are shown above for reference.

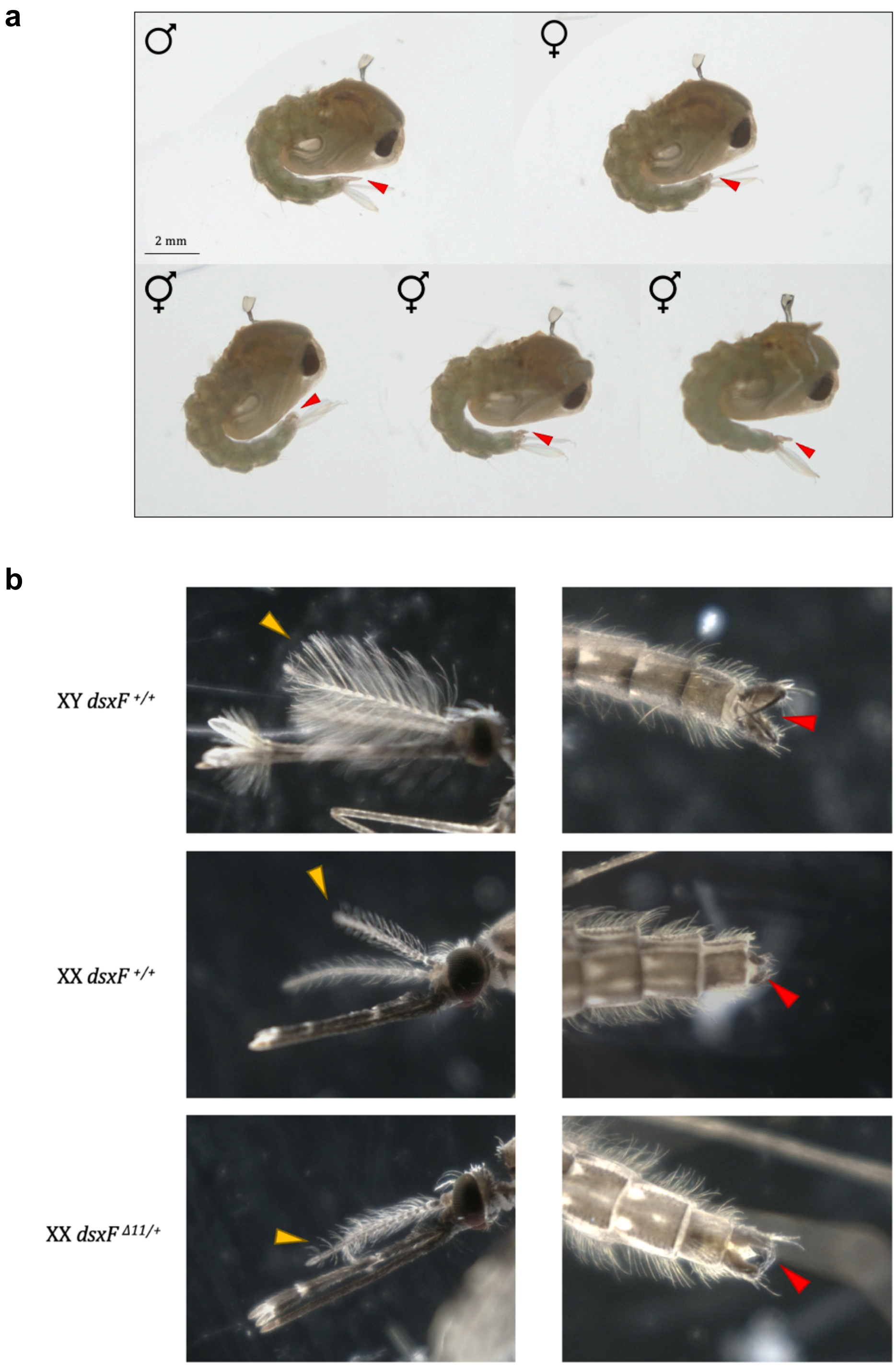

Supplementary figure 3 – Intersex phenotype at pupal and adult stages of chromosomally female individuals heterozygous for the *dsxF*$^{\Delta 11}$ allele. (**a**) Females with one copy of the *dsxF*$^{\Delta 11}$ mutation displayed an intersex phenotype at the pupal stage (bottom row). This was evident in the more developed genital lobe (red arrows) compared to wild-type females (top-right), but less developed than in wild-type males (top-left). (**b**) At adulthood, females heterozygous for *dsxF*$^{\Delta 11}$ (bottom) have female-like pilose antennae (yellow arrows) and a pair of claspers (red arrows) that are dorsally rotated in comparison to the claspers in wild-type males (top), which wild-type females lack (middle).

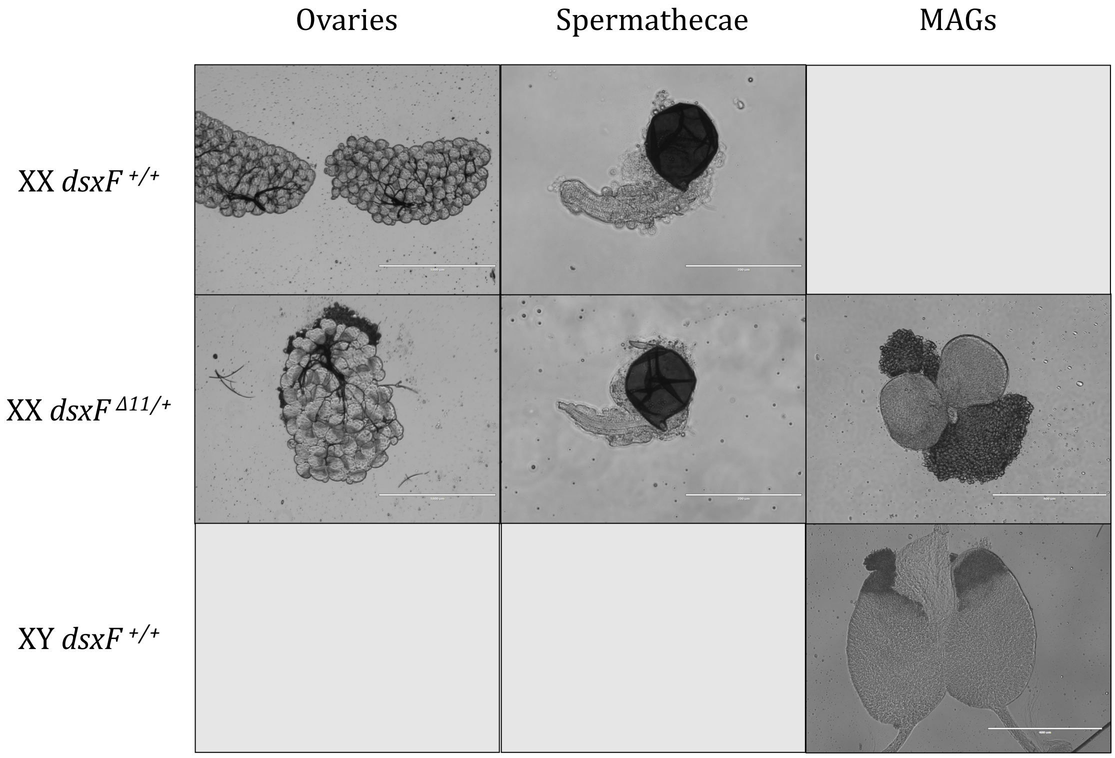

Supplementary figure 4 – Internal organs of chromosomally female individuals with intersex phenotype. Females bearing one copy of the *dsxF*^{Δ11} allele (centre) have ovaries and spermathecae that are visually similar to those of normal females (top). Simultaneously, they have organs resembling male accessory glands (MAGs). The white bar is equivalent to 1000 µm for the ovaries (left), 200 µm for the spermathecae (centre) and 400 µm for the MAGs (right).

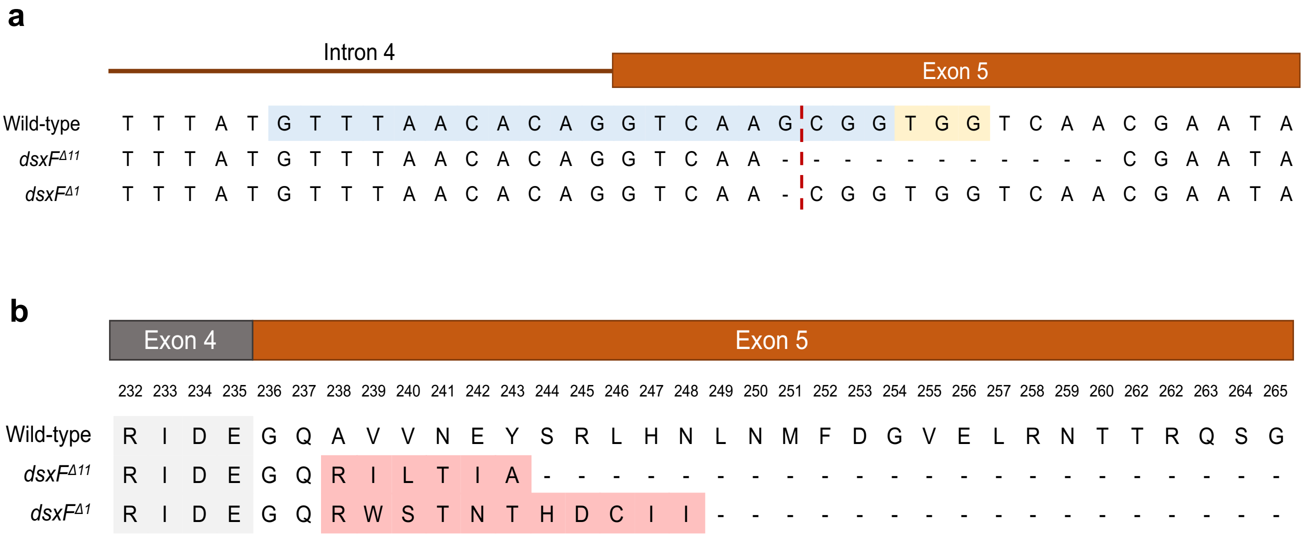

Supplementary figure 5 – Comparison of the nucleotide and amino acid sequences of the two dominant negative mutations identified in the *dsx* locus of *An. gambiae*. (a) Representation of the intron 4 – exon 5 boundary of the *dsx* gene. The gRNA target sequence is highlighted in blue, the PAM at the target site is highlighted in yellow, and a dashed red line shows the cut site. The two dominant mutations identified (the 11bp deletion - *dsxF*$^{\Delta 11}$ - and the 1 bp deletion - *dsxF*$^{\Delta 1}$-) are shown. **(b)** Representation of the amino acid sequence encoded at the end of exon 4 and the coding sequence of exon 5 of the *dsx* gene. The wild-type sequence is shown as a reference. Amino acids that are different from the wild-type reference are highlighted in red.

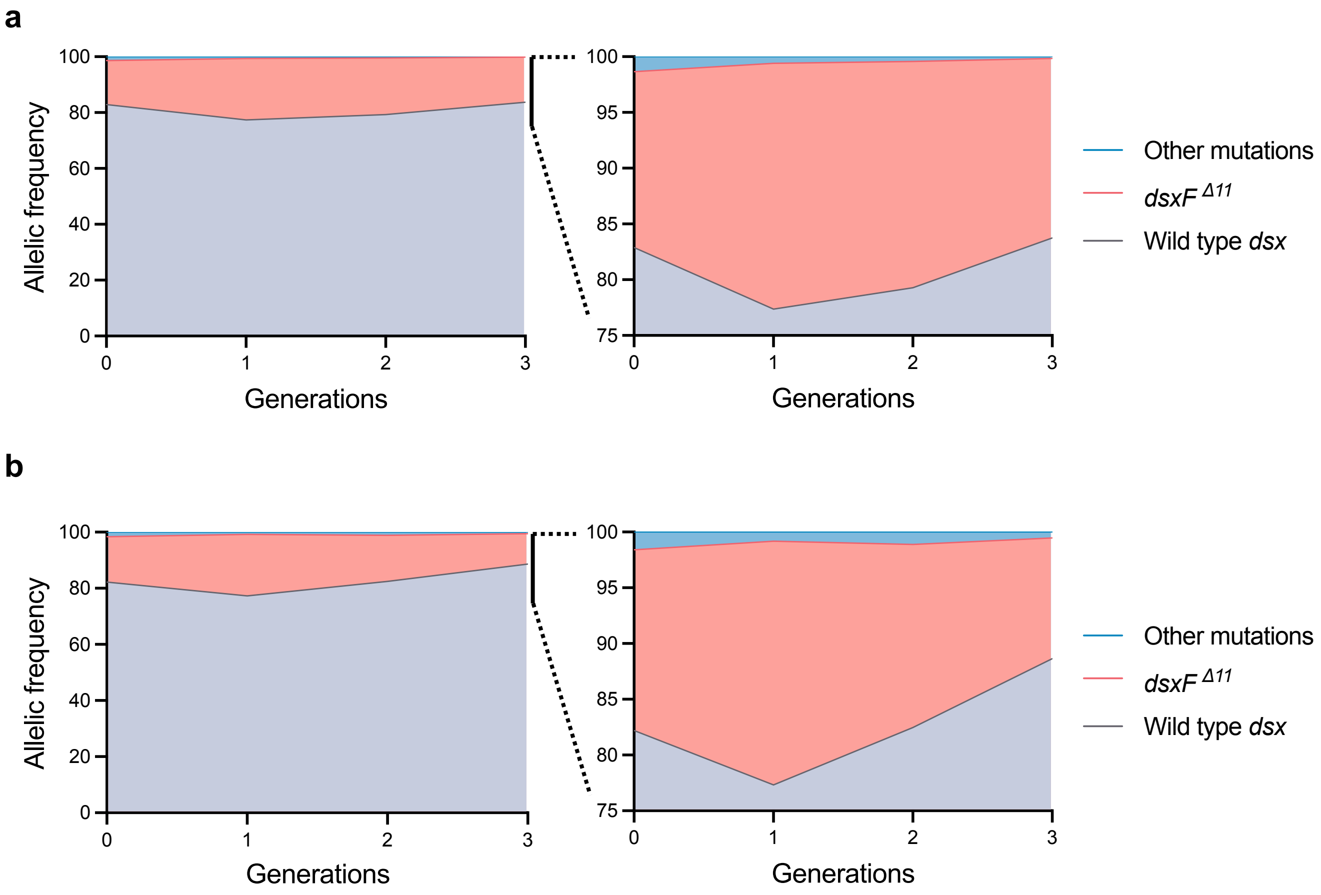

Supplementary figure 6 – Allelic frequency (%) across generations in the caged populations suppression experiment. At every generation, all the adults that formed the caged population were divided into two pools and amplicon sequencing of the *dsx* locus was carried out in each of these pools. The averages of the frequencies of each allele were calculated and displayed in these plots. Here, it is shown the frequency of the released *dsxF*$^{\Delta 11}$ allele (red), the wild-type *dsx* (grey), and the sum of other mutations (blue) across generations in cage 1 (**a**) and in cage 2 (**b**). The plots on the right show a magnification to better understand the dynamics in the frequency of new mutations across time. An estimated 95% of the released YLE^{dsx} males carried the dominant *dsxF*$^{\Delta 11}$ allele. Source data are provided as a Source Data file.

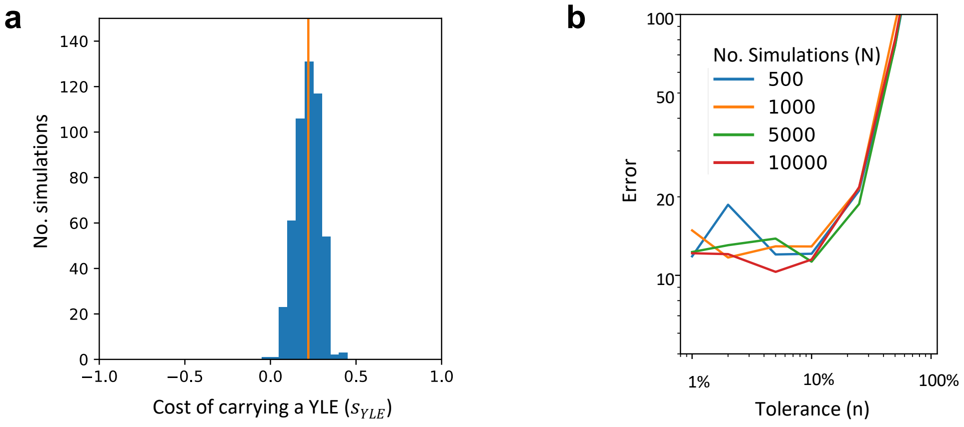

Supplementary figure 7 – Inference of the fitness cost associated to the YLE^{dsx} using an algorithm based on Approximate Bayesian Computation. (**a**) The posterior distribution (blue) of the YLE fitness parameter $sYLE$ with a median of 0.22 (orange) and a 95% credible interval of 0.08-0.33. The algorithm used to obtain the posterior distribution was based on Approximate Bayesian Computation where N=10000 simulations generated with randomly drawn $sYLE$ parameters were compared to the empirical cage trial data and the best fitting *n* = 5% were retained. (**b**) The accuracy and robustness of the estimation to choice of N and n was assessed using cross validation where the error $= \frac{\sum_{i=1}^{499}(\tilde{\theta}_i - \theta_i)^2}{Var(\theta_i)}$, θ_i is the true s_{YLE} parameter used to generate simulation i, $\tilde{\theta}_i$ is the median of the estimated posterior distribution.

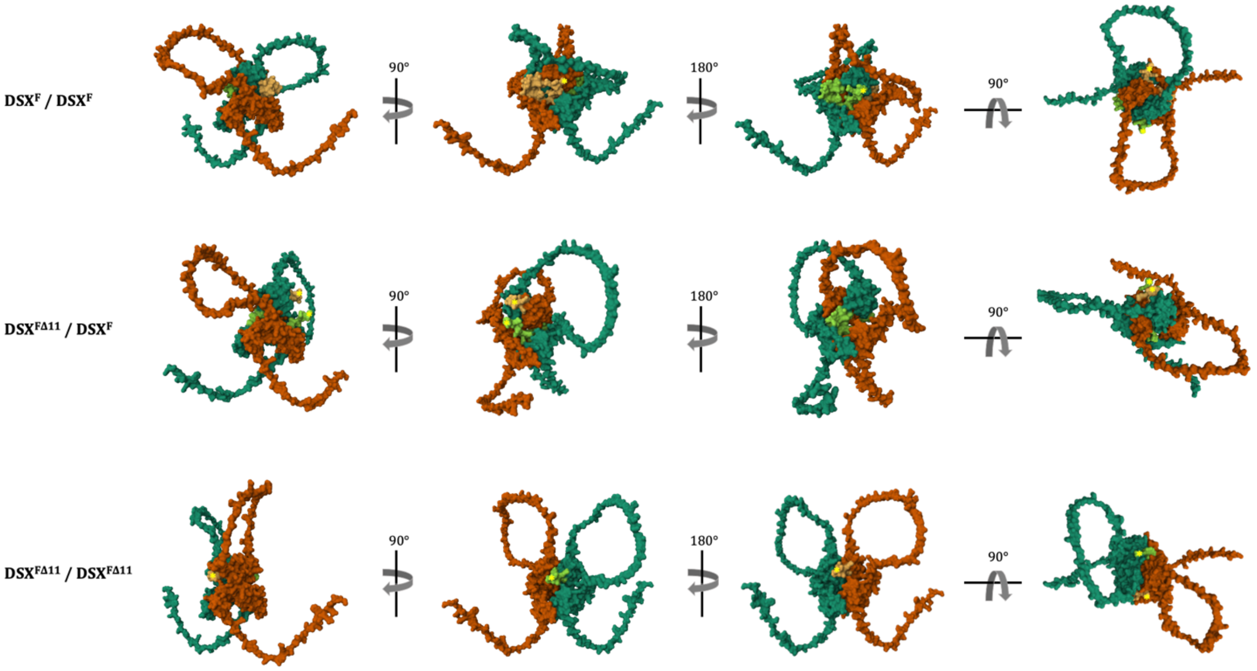

Supplementary figure 8 – Predicted structure by AlphaFold2[1] of the homodimers and heterodimers formed by DSX^{F} and DSX$^{F\Delta11}$ proteins. The top row shows the predicted structure of the wild-type DSX^{F} homodimer (DSX^{F}/DSX^{F}), the middle one illustrates the predicted structure of the heterodimer (DSX^{F}/ DSX$^{F\Delta11}$) and the bottom row shows the predicted structure of the homodimer formed by two DSX$^{F\Delta11}$ proteins (DSX$^{F\Delta11}$/ DSX$^{F\Delta11}$). Monomers are represented in different colours, one in green and one in orange. Lighter colours in the structure illustrate the female specific region of DSX proteins, where the modifications by the *dsxF$^{\Delta11}$* allele are introduced. The C-terminal ends are indicated with a yellow star when shown from each perspective. In the heterodimer, the DSX^{F} protein is represented in green, and the DSX$^{F\Delta11}$ one is shown in orange. While the OD1 domain (half bottom part in the first three columns) is generally conserved among the three dimers, considerable structural changes can be observed in the OD2 and especially in the female specific region of the proteins.

SUPPLEMENTARY TABLES

Supplementary table 1 – Frequencies of new mutations resulting from end joining repair mechanisms at the *dsx* target in the progenies of YLE^{dsx}; *dsxF*$^{GFP\text{-}null/+}$ males. Each row corresponds to the progeny of a different mating couple (A-1 to A-8 are batches of progenies from YLE^{dsx}-A; *dsxF*$^{GFP\text{-}null/+}$ males, and B-1 to B-6 are batches of progenies from YLE^{dsx}-B; *dsxF*$^{GFP\text{-}null/+}$ males). The second column indicates the inheritance bias of the *dsxF*$^{GFP\text{-}null}$ allele (i.e, percentage of the offspring that inherited this allele) as a reference of the homing rates observed in each of these progenies. The third column shows the frequency of all newly generated mutations through end joining repair mechanisms (EJ) in the progeny that were absent in the progenitor male. The fourth column indicates the frequency of the most common mutation found in each progeny.

Progeny	Inheritance of *dsxF*$^{GFP\text{-}null}$ allele (%)	Frequency of new EJ mutations (%)	Frequency of most common mutation (%)
**A-1**	73.33	0.09	0.03
**A-2**	68.50	0.16	0.04
**A-3**	64.10	0.38	0.30
**A-4**	59.46	1.10	1.04
**A-5**	52.38	0.15	0.06
**A-6**	52.38	0.27	0.11
**A-7**	51.51	0.09	0.07
**A-8**	41.46	0.03	0.01
**Average A**	-	**0.29**	**0.21**
**B-1**	89.47	2.89	1.97
**B-2**	89.39	0.53	0.06
**B-3**	83.93	0.58	0.08
**B-4**	77.22	0.20	0.02
**B-5**	50.51	0.04	0.01
**B-6**	48.94	0.01	0.01
**Average B**	-	**0.71**	**0.36**

Supplementary table 2 – Frequencies of new mutations resulting from end joining repair mechanisms at the *dsx* target in the progenies of YLE^{dsx}-A; *dsxF*$^{\Delta 11/+}$ males. Amplicon sequencing was performed in pooled individuals of the progenies of YLE^{dsx}-A; *dsxF*$^{\Delta 11/+}$ males. Each row corresponds to a different set of offspring. The second column indicates the frequency of the *dsxF*$^{\Delta 11}$ allele in the progeny. The third column shows the frequency of the combination of all newly generated mutations through end joining repair mechanisms (EJ) found in each progeny. The fourth column indicates the frequency of the most common mutation (besides *dsxF*$^{\Delta 11}$) in each progeny. Because a wild-type allele will always be inherited from the female, the percentage of individuals bearing each mutation corresponds to the double of the frequency of said mutation (i.e., if the frequency of the *dsxF*$^{\Delta 11}$ allele is 50%, this reflects that 100% of the individuals bear the *dsxF*$^{\Delta 11}$ allele). The sum of all mutations in progeny A-1 is >50%; this might be explained by either contamination or very low loads of nuclease deposition. Only 3 progenies (A-1, A-2, A-4) showed a new mutation in a frequency high enough to represent heterozygous individuals (0.59 – 2.14%).

Progeny	Frequency of *dsxF*$^{\Delta 11}$ allele (%)	Frequency of new EJ mutations (%)	Frequency of most common mutation (%)
**A-1**	49.73	1.58	1.20
**A-2**	46.51	0.72	0.59
**A-3**	46.01	0.27	0.04
**A-4**	45.79	2.81	2.14
**A-5**	44.64	0.34	0.04
**A-6**	43.23	0.44	0.07
**A-7**	40.68	0.39	0.05
**A-8**	37.83	0.17	0.02
**A-9**	37.33	0.29	0.03
**A-10**	36.31	0.20	0.03
**A-11**	35.74	0.22	0.03
**A-12**	26.75	0.11	0.03
**A-13**	25.64	0.17	0.02
**A-14**	24.10	0.16	0.02
**A-15**	23.39	0.13	0.02
**A-16**	17.49	0.09	0.01
**Average**	-	0.51	0.27

Supplementary table 3 – Frequencies of new mutations resulting from end joining repair mechanisms at the *dsx* target in the progenies of YLE^{dsx}-B; *dsxF*$^{Δ11/+}$ males. Amplicon sequencing was performed in pooled females of the progenies of YLE^{dsx}-B; *dsxF*$^{Δ11/+}$ males, separating the ones showing an intersex phenotype from those that had a wild-type phenotype. Each row corresponds to a different set of offspring. The second column shows the frequency of the *dsxF*^{Δ11} allele in the intersex pool, and the third column indicates the frequency of all newly generated mutations through end joining repair mechanisms (EJ) in the male germline and inherited by females showing an intersex phenotype. The fourth column shows the frequency of the wild-type allele in the pool of females with a wild-type phenotype (expected to be 100% unless they inherited a newly generated recessive mutation), and the fifth column indicates the frequency of the aggregate of mutations in this pool. Because a wild-type allele will always be inherited from the female, the percentage of individuals bearing each mutation corresponds to the double of the frequency of said mutation (i.e., if the frequency of the *dsxF*^{Δ11} allele is 50%, this reflects that 100% of the individuals bear the *dsxF*^{Δ11} allele). Only one progeny (B-5) had a different mutation to *dsxF*^{Δ11} in a high enough frequency to represent heterozygous individuals, found in the intersex pool, suggesting that it was a dominant negative mutation. Only one progeny (B-6) had a mutation in high frequency in the pool of wild-type-looking females. They were 2 individuals that were heterozygous for the *dsxF*^{Δ11} allele, suggesting that they had inherited the allele from their progenitor and were either misclassified as wild-type or showed no intersex phenotype at pupal stage. (*) The average of mutations found in the wild-type pool does not include the progeny where the *dsxF*^{Δ11} allele was found (more likely homed than created by MMEJ).

Progeny	Freq of *dsxF*^{Δ11} allele (%) – intersex pool	Freq of new EJ mutations (%) – intersex pool	Freq of wild-type allele (%) – wild-type pool	Freq of new EJ mutations (%) – wild-type pool
**B-1**	50.05	0.05	-	-
**B-2**	50.87	0.07	-	-
**B-3**	51.49	0.07	-	-
**B-4**	50.23	0.12	-	-
**B-5**	37.51	14.26	-	-
**B-6**	50.88	0.06	49.07	50.93
**B-7**	46.51	0.07	99.99	0.01
**B-8**	51.33	0.05	99.99	0.01
**B-9**	51.39	0.06	99.98	0.02
**Average**	-	1.65	-	0.01*

Supplementary table 4 – Model inheritance parameters and values for YLE^{dsx}. Parameters that determine the inheritance of the released dominant mutation, the generation of new end-joining mutations (NHEJ) and sex-ratio distortion. The description of each parameter, the data source, and the values for the modelling of the YLE^{dsx} are displayed.

Parameter	Description	Data source	YLE^{dsx} values
**Gamete production**			
$\boldsymbol{d}$	Proportion of offspring which carry the desired mutation	Experiment	0.945
$\boldsymbol{v}$	Proportion of offspring which carry an alternative mutation	Experiment	0.020
$\boldsymbol{e}$	Probability of homing	$2d-1$	0.890
$\boldsymbol{u}$	Proportion of non-homed chromosomes which are NHEJ	$\frac{\mathrm{v}}{1-d}$	0.455
$\boldsymbol{c}$	Probability of cleavage (in heterozygotes)	$e+(1-e)u$	0.930
$\boldsymbol{j}$	Probability of NHEJ given joining (in heterozygotes)	$\frac{(1-e)u}{e+(1-e)u}$	0.043
$\boldsymbol{\mu}$	Probability of mutation (in wild-type homozygotes)	Unknown	1
$\boldsymbol{p}$	Proportion of dominant NHEJ (in heterozygotes)	Unknown	0
$\boldsymbol{q}$	Proportion of dominant NHEJ (in wild-type homozygotes)	Unknown	0
$\boldsymbol{m}$*	Proportion of Y-bearing sperm produced by males carrying an X-shredder	Pollegioni et al 2020[2]	0.9

*Relevant only when modelling the YLE in the presence of an X-shredder.

Supplementary table 5 – Model fitness parameters. Parameters that determined the fitness effects of the autosomal alleles $\boldsymbol{a}$ and α relative to the WT allele A, and of the X-shredder (XS). The description of each parameter and the values used for each strategy modelled is displayed.

**Fitness costs**		**YLE^{dsx}**	**YLE^{dsx} + XS**	**SIT and RIDL**	**fsRIDL**	**XS**
s_{α}^{G}	Fitness cost for the dominant cleavage resistant allele	1^{F}	1^{F}	1	1^{F}	-
s_{a}^{G}	Fitness cost for the recessive cleavage resistant allele	1^{F}	1^{F}	-	-	-
h_{α}^{G}	Dominance coefficient for the dominant cleavage resistant allele	1^{F}	1^{F}	1	1^{F}	-
h_{a}^{G}	Dominance coefficient for the recessive cleavage resistant allele	0	0	-	-	-
$s_{a\alpha}^{G}$	Fitness cost for heterozygous for the two types of cleavage resistant allele	1^{F}	1^{F}	-	-	-
s_{XS}^{M}	Fitness cost for the X-shredder allele in males	-	0.2*	-	-	0

The superscript ^{F} indicates the case where fitness costs are applied only to females and the value in males is zero.

*Pollegioni et al 2020[2].

SUPPLEMENTARY METHODS

Mathematical modelling

Gamete transmission

In males carrying the YLE, the probability of gamete transmission can be altered due to cleavage of the WT autosomal target. Cleavage of the WT allele occurs with probability c, and, in WT heterozygotes repair of the cleaved chromosome occurs by non-homologous end-joining with probability j, converting the WT A allele to a cleavage-resistant allele, a or α, with probability p and $1-p$ respectively. Alternatively, the cleaved chromosome is repaired through homology-directed repair, converting the A allele to either an a or α allele, depending on the alternative allele present in the genotype. In males carrying the YLE and two copies of the WT autosomal allele, cleavage and mutation of each WT allele occurs with probability μ, converting the A allele to a cleavage-resistant allele, a or α, with probability q and $1-q$ respectively. Since the editor is located on the Y chromosome, there is no change in gamete transmission due to YLE activity in females. Haploid gamete genotypes are generated assuming random segregation of the edited (or unedited) parental diploid genotype, and the proportion of zygotes of each diploid genotype is calculated assuming random pairing of of male and female gametes. To model the effects of the autosomal X-shredder, males carrying at least one b allele produce Y and X bearing sperm at a ratio of $m : 1-m$. When m is equal to 1 all gametes produced by males carrying the b allele contain a Y chromosome (i.e. all offspring fertilised by these sperm are male) whereas when m is equal to 0.5 there is no sex-ratio distortion. The influence of the YLE and the edits it makes on gamete transmission can be summarised in the matrix I where $I^{X}_{i,j,k}$ is the expected proportion of zygote genotype k of sex X produced by a mother with genotype $i,$ and father with genotype j and can be found in the GitHub repository. Descriptions of all inheritance parameters and their values when modelling the YLE^{dsx} can be found in Supp. Table 4. Note that for SIT, RIDL, fsRIDL, and the X-shredder alone no editing occurs, therefore c, j, μ, p and q were equal to 0. When modelling strategies with an idealised autosomal X-shredder m was equal to 1.

Fitness effects

The fitness of individuals can be affected by the presence of alleles which disrupt the function of the A locus. To model this the fitness of A/A individuals is normalised to 1;

and the relative fitness of genotypes homozygous for the dominant mutation α/α is $1 - s_{\alpha}^{G}$; homozygous for the recessive mutation a/a is $1 - s_{\mathrm{a}}^{G}$; heterozygous for the dominant mutation and WT A/α is $1 - s_{\alpha}^{G} h_{\alpha}^{G}$; heterozygous for the recessive mutation and WT A/a is $1 - s_{\mathrm{a}}^{G} h_{\mathrm{a}}^{G}$; and heterozygous for the dominant and recessive mutation a/α is $1 - s_{\mathrm{a}\alpha}^{G}$. Here, s_{α}^{G}, s_{a}^{G}, and $s_{\mathrm{a}\alpha}^{G}$ are selection coefficients, and h_{α}^{G} and h_{a}^{G} are dominance coefficients, each of which were allowed to vary depending on sex (G). Parameterisation in this way allows for modelling of female-specific fitness effects of the YLE and comparator strategies such as the fsRIDL, as well as bi-sex fitness effects such as for in SIT and RIDL. For simplicity, it was assumed that the A locus is essential for survival to reproductive maturity. We also include a male-specific cost associated with carrying the YLE where the survival of these males from pupae to reproductive maturity is multiplied by $1 - s_{\mathrm{XS}}^{M}$.

Small and large cage trials of *An. gambiae* males carrying an X-shredder revealed two fitness effects of the X-shredder: reduced fertility in males carrying an X-shredder and reduced survival of daughters with X-shredder fathers due to inheriting X chromosomes exposed to the X-shredder[2,3]. For simplicity, we incorporate only the first cost since it effects all X-shredder males and is expected to be the most impactful, whereas the second cost is restricted to the rare females produced from X-shredder males. Since we assume random mating and that the number of males is not limiting to the number of eggs fertilised, reduced male fertility is modelled by i) reducing the survival of males from pupae to adult by a factor of $1 - s_{\mathrm{XS}}^{M}$, where s_{XS}^{M} is the fitness cost of the X-shredder males and ii) reducing the number of eggs laid by all females by a factor of $1 - s_{\mathrm{XS}}^{M} N_{p}^{M}$, where N_{p}^{M} is the proportion of X-shredder carrying males in the population at the pupal stage. For simplicity we assume the fitness costs are the same whether males carry one or two X-shredder constructs. Descriptions of the fitness parameters and their values for each strategy can be found in Supp. Table 5.

Population biology in the deterministic model

To simulate the impact of releasing the construct(s) into a wild population we use a deterministic model (based on the model developed by Burt and Deredec[4]) which simulates a single, infinite-sized population where mating occurs randomly, with discrete, non-overlapping generations and three life stages (larvae, pupae and adults).

In each generation females produce f eggs which are fertilised by males assuming random mating and that the number of males is not limiting to the number of eggs fertilised. Larval survival to the pupal stage is density-dependent according to the Beverton-Holt model, where the probability of survival is $\theta_j \frac{\beta}{\beta+N_j}$, θ_j is the density-independent probability that a juvenile survives to adulthood, β determines the strength of density-dependent mortality and N_j is the total number of juveniles in the population. In this study all results are reported as relative population sizes of fertile females compared to the starting population at equilibrium, therefore they are not affected by the value of β. The intrinsic rate of increase (R_m) of the wild-type population is $\frac{f\theta_j}{2}$. A value of 6 was used for the R_m in all simulations unless otherwise stated[5]. Depending on the strategy being modelled, the genotype-dependent fitness costs are either applied before density-dependent mortality (as if larvae die before reaching pupation, as in SIT) or after (as if pupae die before maturing into reproductively active adults, as in RIDL, fsRIDL and YLE, or never reach reproductive maturity due to infertility. When modelling the YLE^{dsx}, released males were assumed to carry a YLE construct and a single copy of a dominant female-specific cleavage-resistant allele (A/α). In the case of SIT, RIDL, fsRIDL and the X-shredder alone, released males carried a WT Y-chromosome (Y) and were homozygous for the fitness inducing modification (α), the fitness effects of which varied depending on the strategy being modelled. The population was censused at the zygote stage where autosomal allele frequencies were calculated in males and females independently and averaged, whereas frequencies of Y-linked alleles refer only to males. Estimated release rates required were accurate to 3 decimal places.

Population biology in the stochastic model

To simulate a cage population, we modelled a population of finite size in discrete generations, mirroring the design of the experimental cage trial. Each simulation began with 300 WT male and 300 WT female pupae. In the first-generation 300 YLE carrying male pupae were added to the population. Next the number of mating adults ($A_i^X(t)$) of each sex X and sex-specific genotype i in generation t was sampled from a binomial distribution, $A_i^X(t) \sim Bin(P_i^X(t), M)$, where $P_i^X(t)$ is the number of pupae of X sex and genotype i in generation t and M is the probability of pupae emerging and

reaching reproductive maturity. We next applied genotype-specific fitness costs to females, assuming those carrying at least one copy of the dominant mutation or two copies of either a recessive or dominant mutation at the *dsx* locus did not enter the mating pool. To allow males carrying a YLE to have reduced or enhanced mating competitiveness compared to the WT the number of WT males were multiplied by $1 - s_{YLE}$, before the frequency of mating males of each genotype was calculated. Where $s_{YLE} < 0$ YLE-bearing males have enhanced competitiveness compared to the WT whereas when $s_{YLE} > 0$ it is reduced. To simulate mating the number of mated adult females of genotype i mated to males of each genotype $1 \ldots j$ in generation t was $A^{FM}_{i,1\ldots j}(t) \sim M(A^F_i(t), a^M_{1\ldots j}(t))$ where $A^F_i(t)$ is the number of mated adult females of genotype i in generation t and $a^M_{1\ldots j}$ is the frequency of males of each of the twelve male genotypes (1 to j). Here we assumed males and females mated at random, that each female mated only once, that males could mate multiple females and that the number of males was not limiting to the formation of mating pairs. The number of zygotes of genotype $(1 \ldots k)$ produced by each mated female was calculated by sampling from a multinomial distribution, $Z^X_{i,j,1\ldots k} \sim M(o, I^X_{i,j,1\ldots k})$ where o is the number of eggs produced by the focal mated female, drawn from a vector of empirically observed egg numbers (source data provided as a Source Data file), and $I^X_{i,j,k}$ is the expected proportion of zygotes produced by each mating pair. The number of zygotes surviving to pupation of each genotype i at generation $t + 1$ was then $P^X_i(t + 1) \sim Bin(Z^X_i(t + 1), H\,L)$, where $P^X_i(t + 1)$ is the number of pupae of X sex and genotype i in generation $t + 1$, H is the probability of an egg hatching and L is the probability of surviving to pupation. 600 pupae were chosen at random without replacement to seed the next generation and were used to calculate the numbers of individuals carrying a YLE construct and the number of WT females.

Estimating YLE fitness effects using Approximate Bayesian Computation

We next compared the empirical cage trial results to the simulations and estimated the potential fitness effect of the YLE on males carrying it using a simple algorithm based on Approximate Bayesian Computation. Since the empirical data suggested there was no impact on the number of eggs produced by females mated to YLE-males, we

estimated fitness effects that may enhance or reduce mating competitiveness (s_{YLE}). To estimate s_{YLE}:

- A parameter was drawn from uniform prior between -1 and 1.
- Since the empirical data contained two trials, two simulations were generated where s_{YLE} was set equal to the drawn parameter.
- The Euclidian distance was calculated between the frequency of YLE carriers in the population observed over time in the cage experiment and the simulation.
- The first three steps were repeated N times.
- A posterior distribution for s_{YLE} was obtained by collecting the s_{YLE} parameters used to generate simulations where the distance metric is in the lowest n quantile (Supp. Fig. 9a).
- The point estimate was obtained by taking the median of the posterior distribution and the 95% credible interval calculated.

We also performed cross validation following the method described by Csilléry et al.[6] (Supp. Fig. 9b), to evaluate the accuracy and robustness of the estimate to our choice of n and N. To do so the ith simulation was randomly selected as a validation simulation. This was removed from the simulated dataset and the posterior distribution was inferred using the remaining simulations as a test set. This was repeated 499 times, and the overall error calculated using

$$Error = \frac{\sum_{i=1}^{499}\left(\tilde{\theta}_i - \theta_i\right)^2}{Var(\theta_i)},$$

where θ_i is the true s_{YLE} parameter used to generate simulation i and $\tilde{\theta}_i$ is the median of the estimated posterior distribution.

This was repeated for values of N between 500 and 10000 and n between 1% and 90%. The final inference was performed using N=10000 and n=5%.

SUPPLEMENTARY REFERENCES

1. Mirdita, M. *et al.* ColabFold: making protein folding accessible to all. *Nat Methods* **19**, 679–682 (2022).
2. Pollegioni, P. *et al.* Detecting the population dynamics of an autosomal sex ratio distorter transgene in malaria vector mosquitoes. *Journal of Applied Ecology* **57**, 2086–2096 (2020).
3. Galizi, R. *et al.* A synthetic sex ratio distortion system for the control of the human malaria mosquito. *Nat Commun* **5**, 3977 (2014).
4. Burt, A. & Deredec, A. Self-limiting population genetic control with sex-linked genome editors. *Proceedings of the Royal Society B: Biological Sciences* **285**, 20180776 (2018).
5. Deredec, A., Godfray, H. C. J. & Burt, A. Requirements for effective malaria control with homing endonuclease genes. *Proceedings of the National Academy of Sciences* **108**, E874–E880 (2011).
6. Csilléry, K., François, O. & Blum, M. G. B. abc: an R package for approximate Bayesian computation (ABC). *Methods in Ecology and Evolution* **3**, 475–479 (2012).
